# Kinetic Study on the Reactivity of Azanone (HNO) toward Cyclic *C*-Nucleophiles

**DOI:** 10.3390/ijms222312982

**Published:** 2021-11-30

**Authors:** Angelika Artelska, Monika Rola, Michał Rostkowski, Marlena Pięta, Jakub Pięta, Radosław Michalski, Adam Bartłomiej Sikora

**Affiliations:** Institute of Applied Radiation Chemistry, Lodz University of Technology, Zeromskiego 116, 90-924 Lodz, Poland; angelika.artelska@dokt.p.lodz.pl (A.A.); monika.rola@dokt.p.lodz.pl (M.R.); michal.rostkowski@p.lodz.pl (M.R.); marlena.pieta@p.lodz.pl (M.P.); jakub.pieta@p.lodz.pl (J.P.); radoslaw.michalski@p.lodz.pl (R.M.)

**Keywords:** azanone, *C*-nucleophiles, Angeli’s salt, boronate probe, peroxynitrite

## Abstract

Azanone (HNO) is an elusive electrophilic reactive nitrogen species of growing pharmacological and biological significance. Here, we present a comparative kinetic study of HNO reactivity toward selected cyclic *C*-nucleophiles under aqueous conditions at pH 7.4. We applied the competition kinetics method, which is based on the use of a fluorescein-derived boronate probe FlBA and two parallel HNO reactions: with the studied scavenger or with O_2_ (*k* = 1.8 × 10^4^ M^−1^s^−1^). We determined the second-order rate constants of HNO reactions with 13 structurally diverse *C*-nucleophiles (*k* = 33–20,000 M^−1^s^−1^). The results show that the reactivity of HNO toward *C*-nucleophiles depends strongly on the structure of the scavenger. The data are supported with quantum mechanical calculations. A comprehensive discussion of the HNO reaction with *C*-nucleophiles is provided.

## 1. Introduction

The discovery of important physiological function of the nitric oxide (^•^NO) as a signaling agent in mammals [1] has established a new paradigm in physiology and medicine [2]. Over the past thirty years, several other small, reactive molecules have been identified as having similar signaling properties. This class of signaling molecules includes nitric oxide, carbon monoxide (CO), hydrogen sulfide (H_2_S) and azanone (HNO, commonly known as nitroxyl) [3,4]. Formally, the protonated product of one-electron reduction of nitric oxide, HNO is an elusive reactive electrophilic nitrogen species of growing pharmacological importance [5,6,7,8]. Interest in azanone donors and the biological chemistry of HNO has increased significantly in recent years, mainly due to the positive effects of HNO on the vascular system and the possible applicability of its donors as therapeutics in the treatment of heart failure. Research on azanone reactivity is hindered by rapid spontaneous HNO dimerization (*k* = 8 × 10^6^ M^−1^s^−1^) [9], resulting in the formation of hyponitrous acid, which subsequently dehydrates to nitrous oxide and water (reaction 1).

2 HNO → [HONNOH] → N_2_O + H_2_O (1)

Due to the rapid dimerization of HNO, in both chemical and biological research on azanone properties, it is necessary to use HNO donors that release azanone in a controlled manner [10]. The most commonly used HNO donor in biological studies is Angeli’s salt (Na_2_N_2_O_3_), which has been known since 1896 [11]. Piloty’s acid, another HNO donor, was also reported the same year [12]. Several classes of compounds able to spontaneously release HNO under physiological conditions have been described in the past 25 years. These include derivatives of Piloty’s acid [13,14,15,16], primary amine-based diazeniumdiolates [17,18,19,20], acyloxy nitroso compounds [21,22,23], and *N*-substituted hydroxylamines with a carbon-based leaving group [24,25,26,27]. The latter class of HNO donors was designed, synthesized, studied and described by Toscano and coworkers. In their initial report, they described new azanone donors based on Meldrum’s acid, barbituric acid and pyrazolone that release HNO with high efficiency. Further studies of barbituric acid- and pyrazolone-based HNO donors showed that these compounds produce azanone under physiological conditions with half-lives spanning from minutes to days. It has been also shown that HNO reacts with pyrazolones (*k*~8 × 10^5^ M^−1^s^−1^), forming the corresponding *N*-substituted hydroxylamines [24]. It has been suggested that this reaction could be a useful route to synthesize azanone donors [24].

During the last two decades, there has been great progress in the understanding of azanone chemistry and the chemical biology. However, HNO remains the most elusive nitrogen species and its reactivity has not been described well in terms of kinetics. To the best of our knowledge, there are no reports in the literature on the reactivity of HNO towards *C*-nucleophiles (with the exception of the studies by Toscano, mentioned above). This study fills that gap.

**Scheme 1:**
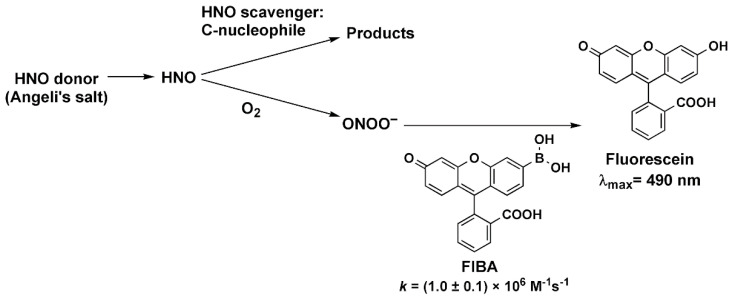
Reaction model used to determine the rate constants of the reactions between HNO and the studied HNO scavengers using the competition kinetic approach.

In this study, we applied the competition kinetics method described previously [28,29,30] to determine the reactivity of HNO towards selected *C*-nucleophiles in aqueous solutions at physiological pH. We chose pH 7.4 to allow a direct comparison of the rate constants of HNO reactions with *C*-nucleophiles with the values determined previously for other azanone scavengers. HNO released from Angeli’s salt reacts with molecular oxygen and *C*-nucleophile, when it is present. Reaction of HNO with molecular oxygen results in the formation of peroxynitrite (ONOO^−^) [28], which can be easily detected with the use of a boronate probe [31,32,33,34,35]. We used the novel fluorescein-based monoboronate probe FlBA, which reacts rapidly and directly with peroxynitrite (*k*_FlBA_ = 1 × 10^6^ M^−1^s^−1^, pH 7.4, 25 °C; for details of the synthetic procedure, spectroscopic and kinetic data see the Supplementary Materials), to form fluorescein (FlOH), that can be easily monitored by UV-vis spectroscopy or with the use of spectrofluorimeter. The reactivity of FlBA probe towards peroxynitrite (see Figure S2) and hydrogen peroxide (see Figure S3) is similar to the reactivity of another fluorescein-derived boronate probe, FlBE [32,36], and is typical for arylboronates reaction with peroxynitrite [32,33,34,35]. A reaction model illustrating the applied competition kinetic method is presented in Scheme 1.

The rate of fluorescein accumulation over time is expressed by Equation (2),

(2) $$v=\frac{d\left[\mathrm{FlOH}\right]}{dt}=k_{\mathrm{FlBA}}\left[\mathrm{FlBA}\right]\left[{\mathrm{ONOO}}^{-}\right]$$

where *k*_FlBA_ is the rate constant for the FlBA reaction with peroxynitrite. Changes in the concentrations of HNO and peroxynitrite over time are expressed by Equations (3) and (4),

(3) $$\frac{d\left[\mathrm{HNO}\right]}{dt}=k_{\mathrm{AS}}\left[\mathrm{AS}\right]-k_{\mathrm{nucleophile}}\left[\mathrm{nucleophile}\right]\left[\mathrm{HNO}\right]-k_{O_{2}}\left[O_{2}\right]\left[\mathrm{HNO}\right]$$

(4) $$\frac{d\left[{\mathrm{ONOO}}^{-}\right]}{dt}=k_{O_{2}}\left[O_{2}\right]\left[\mathrm{HNO}\right]-k_{\mathrm{FlBA}}\left[\mathrm{FlBA}\right]\left[{\mathrm{ONOO}}^{-}\right]$$

where *k*_AS_ is the rate constant of Angeli’s salt decomposition, and *k*_nucleophile_ and *k*_O2_ are the rate constants of HNO reaction with *C*-nucleophile and molecular oxygen, respectively. To solve the above equations, a steady state approximation was made (Equations (5) and (6)).

(5) $$\frac{d\left[\mathrm{HNO}\right]}{dt}=0$$

(6) $$\frac{d\left[{\mathrm{ONOO}}^{-}\right]}{dt}=0$$

The solution of aforementioned equations leads to Equations (7) and (8).

(7) $$\left[\mathrm{HNO}\right]=\frac{k_{\mathrm{AS}}\left[\mathrm{AS}\right]}{k_{\mathrm{nucleophile}}\left[\mathrm{nucleophile}\right]+k_{O_{2}}\left[O_{2}\right]}$$

(8) $$\left[{\mathrm{ONOO}}^{-}\right]=\frac{k_{O_{2}}\left[O_{2}\right]}{k_{\mathrm{FlBA}}\left[\mathrm{FlBA}\right]}\left[\mathrm{HNO}\right]=\frac{k_{\mathrm{AS}}\left[\mathrm{AS}\right]}{k_{\mathrm{FlBA}}\left[\mathrm{FlBA}\right]}\cdot \frac{k_{O_{2}}\left[O_{2}\right]}{k_{\mathrm{nucleophile}}\left[\mathrm{nucleophile}\right]+k_{O_{2}}\left[O_{2}\right]}$$

The rate of fluorescein formation in the presence of *C*-nucleophile is expressed by Equation (9), whereas, in the absence of that HNO scavenger, it is expressed by Equation (10).

(9) $$v_{i}=k_{\mathrm{AS}}\left[\mathrm{AS}\right]\cdot \frac{k_{O_{2}}\left[O_{2}\right]}{k_{\mathrm{nucleophile}}\left[\mathrm{nucleophile}\right]+k_{O_{2}}\left[O_{2}\right]}$$

(10) $$v_{0}=k_{\mathrm{AS}}\left[\mathrm{AS}\right]$$

Comparison of those equations results in Equation (11).

(11) $$\frac{v_{0}}{v_{i}}-1=\frac{k_{\mathrm{nucleophile}}}{k_{O_{2}}}\cdot \frac{\left[\mathrm{nucleophile}\right]}{\left[O_{2}\right]}$$

In our study, we decided to monitor fluorescein formation with the use of UV-Vis spectrophotometry to demonstrate that the kinetics of HNO reactions with its scavengers can be studied with the use of very basic equipment. It can be also done by fluorescence measurements, as we have shown previously for the PC1 and CBA boronate probes [28,29,30].

## 2. Results and Discussion

**Figure 1:**
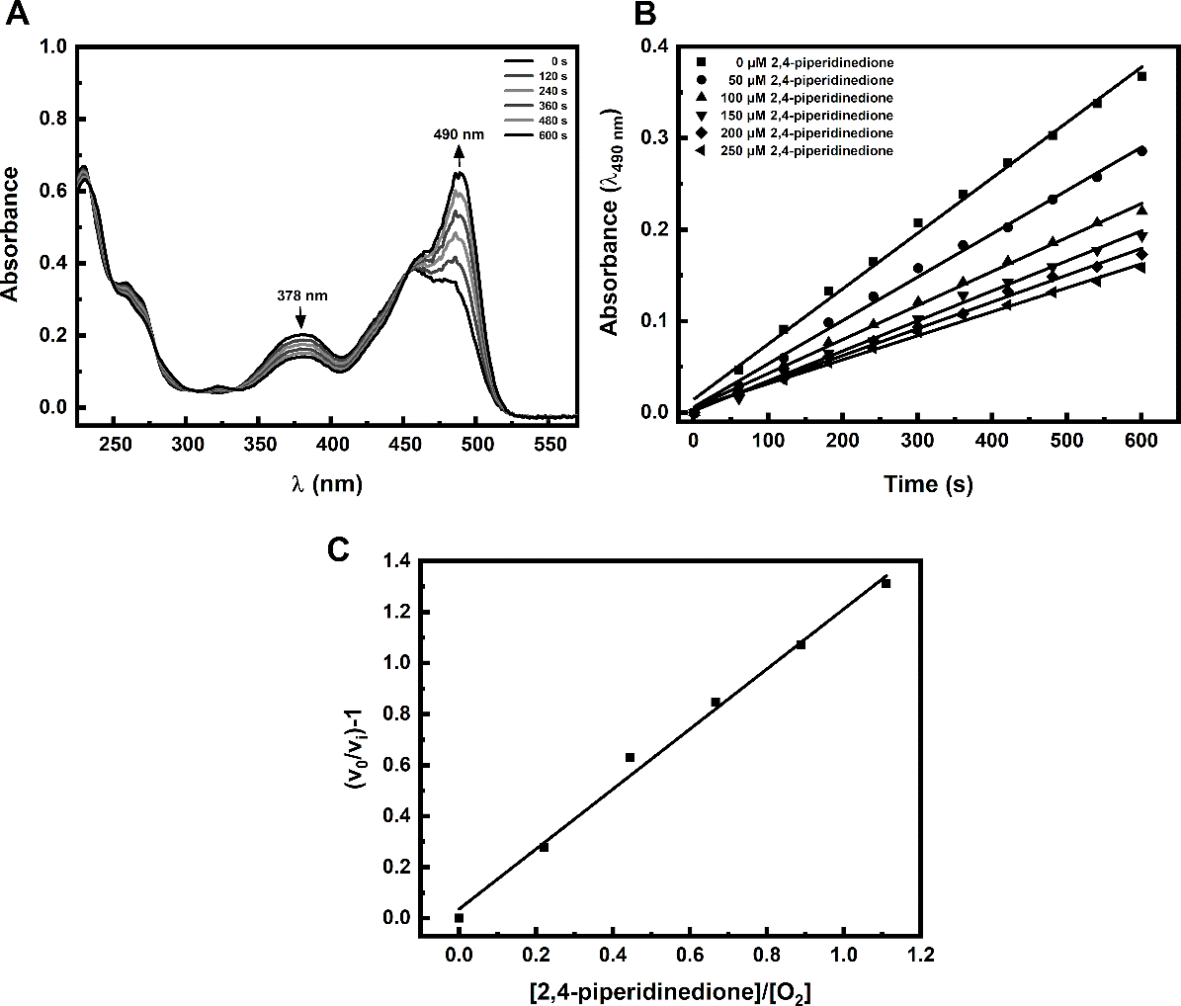
(**A**) Changes in the absorption spectrum due to oxidation of a boronate probe (FlBA) in an aerated aqueous solution of Angeli’s salt in the absence of HNO scavengers. (**B**) An example of the effect of an HNO scavenger 2,4-piperidinedione on the initial rate of fluorescein formation from the FlBA boronate probe. (**C**) Example plot of (*v*_0_/*v*_i_) − 1 versus the [S]/[O_2_] ratio. The reaction mixture contained: 25 µM FlBA, 20 µM Angeli’s salt, 2,4-piperidinedione (0–250 µM), phosphate buffer (pH 7.4, 50 mM), dtpa (100 µM). Each solution contained 5% (vol.) CH_3_CN. The concentration of molecular oxygen was assumed to be 225 µM. Measurements were carried out at 25 °C.

**Scheme 2:**
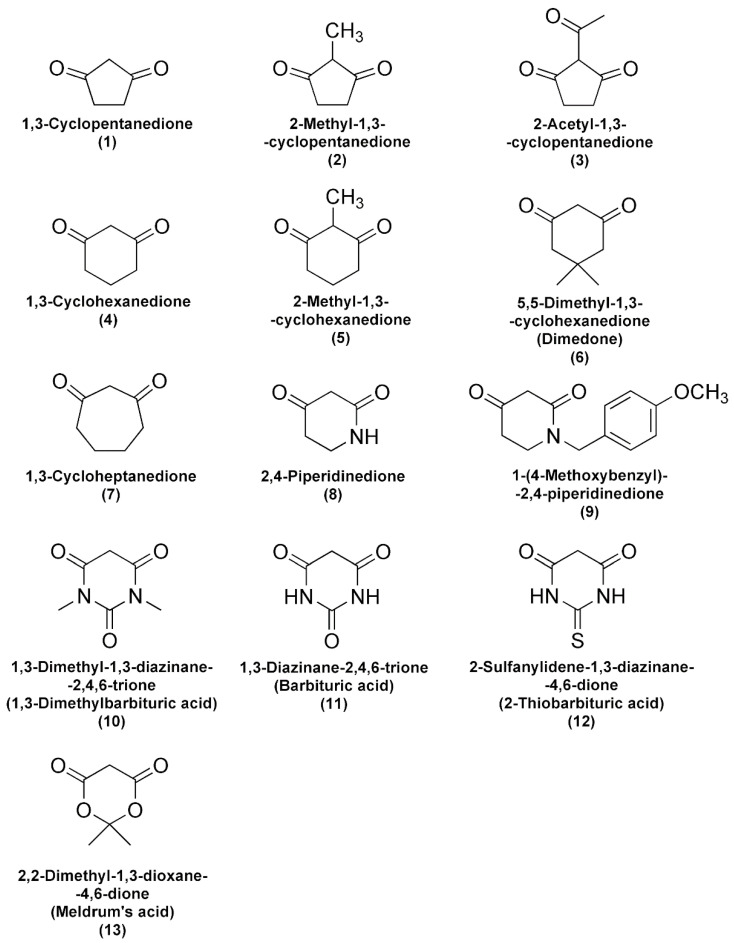
Chemical structures of cyclic *C*-nucleophiles used in this study.

Incubation of FlBA boronate probe in aerated aqueous solution of Angeli’s salt resulted in oxidation of the probe to fluorescein. As shown in Figure 1A the decomposition of Angeli’s salt is accompanied by fluorescein formation as reflected in the disappearance of the absorption bands of boronate probe at 378 nm and the build-up of absorption of fluorescein at 490 nm. In the presence of an HNO scavenger that oxidation is inhibited in a concentration dependent manner (Figure 1B). The rate constant for the reaction of HNO with the scavenger can be determined based on the slope of the plot of (*v*_0_/*v*_i_) − 1 versus the [nucleophile]/[O_2_] ratio (Figure 1C). The second-order rate constant for the reaction of HNO with O_2_ was determined previously to be equal (1.8 ± 0.3) × 10^4^ M^−1^s^−1^ [28]. Using the FlBA probe, we determined the second order rate constants of HNO reactions with selected structurally diverse *C*-nucleophiles (*k* = 33–20,000 M^−1^s^−1^). The chemical structures of the studied *C*-nucleophiles are presented in Scheme 2. The determined rate constants are summarized in Table 1.

Quantum mechanical calculations were performed in order to better understand the structure–reactivity relationship in the reaction of cyclic *C*-nucleophiles with HNO. Reaction energy pathways were followed from separated *C*-nucleophile anions and HNO to the product of *C*-nucleophile addition to the N=O double bond. The calculated energy barriers of HNO reactions with *C*-nucleophiles vary from 41.34 kJ/mol in the case of highly reactive 1-(4-methoxybenzyl)-2,4-piperidinedione (**9**) to 90.93 kJ/mol in the case of the least reactive 2-acetyl-1,3-cyclopentanedione (**3**). The results of quantum mechanical calculations are summarized in Table 1 and discussed below together with the results of the kinetic studies.

Our results show that the reactivity of HNO toward *C*-nucleophiles strongly depends on the structure of the scavenger. First, we examined the effect of *C*-nucleophile ring size on reactivity towards azanone. We selected three cyclic *C*-nucleophiles: 1,3-cyclopentanedione (**1**), 1,3-cyclohexanedione (**4**), and 1,3-cycloheptanedione (**7**) (Scheme 2). The obtained second order rate constants show an increase in *C*-nucleophile reactivity toward HNO with increasing ring size (*k*_1_ = 2.8 × 10^2^ M^−1^s^−1^, *k*_4_ = 2.2 × 10^3^ M^−1^s^−1^, *k*_7_ = 6.8 × 10^3^ M^−1^s^−1^). The observed rate constants are eightfold and 24-fold higher compared to 1,3-cyclopentanedione (**1**). The corresponding calculated energy barriers are equal to 72.53, 59.28 and 57.52 kJ/mol, for 1,3-cyclopentanedione (**1**), 1,3-cyclohexanedione (**4**) and 1,3-cycloheptanedione (**7**), respectively.

The methylation of α-carbon results in a marked increase in the corresponding second order rate constants. In the cases of 1,3-cyclopentanedione (**1**) and 2-methyl-1,3-cyclopentanedione (**2**) (*k*_1_ = 2.8 × 10^2^ M^−1^s^−1^, *k*_2_ = 3.2 × 10^3^ M^−1^s^−1^) as well as 1,3-cyclohexanedione (**4**) and 2-methyl-1,3-cyclohexanedione (**5**) (*k*_4_ = 2.2 × 10^3^ M^−1^s^−1^, *k*_5_ = 1.1 × 10^4^ M^−1^s^−1^), we observed 11-, and fivefold increases. This effect can be attributed simply to the electronic effect of an electron donating group. The corresponding calculated energy barriers for the reaction of 2-methyl-1,3-cyclopentanedione (**2**) and 2-methyl-1,3-cyclohexanedione (**5**) are equal to 57.92 and 50.03 kJ/mol, respectively.

The acylation of α-carbon in 1,3-cyclopentanedione has the opposite effect. We were only able to evaluate the second order rate constant of the reaction of 2-acetyl-1,3-cyclopentanedione (**3**) with HNO, based on the effect of 10 mM 2-acetyl-1,3-cyclopentanedione (**3**) on FlBA oxidation. The corresponding calculated energy barrier for the reaction of 2-acetyl-1,3-cyclopentanedione (**3**) with HNO is equal to 90.93 kJ/mol. The alkylation of 1,3-cyclohexanedione (**4**) at the C-5 position has no effect on *C*-nucleophile reactivity (*k*_4_ = 2.2 × 10^3^ M^−1^s^−1^, *k*_6_ = 2.5 × 10^3^ M^−1^s^−1^, which equates to only about 0.8 kJ/mol difference in computed energy barriers).

Next, we studied the reactivity of two cyclic *C*-nucleophiles containing one heteroatom in the ring: 2,4-piperidinedione (**8**) and 1-(4-methoxybenzyl)-2,4-piperidinedione (**9**). The obtained second-order rate constants show an increase in *C*-nucleophile reactivity toward HNO, compared to 1,3-cyclohexanedione (**4**) (*k*_4_ = 2.2 × 10^3^ M^−1^s^−^^1^, *k*_8_ = 2.0 × 10^4^ M^−1^s^−1^, *k*_9_ = 1.4 × 10^4^ M^−1^s^−1^). The rate constants of the two cyclic *C*-nucleophiles increased ninefold and sixfold, respectively. The corresponding calculated energy barriers for the reaction of 2,4-piperidinedione (**8**) and 1-(4-methoxybenzyl)-2,4-piperidinedione (**9**) are equal to 45.65 kJ/mol and 41.34 kJ/mol, respectively.

We also studied the reactivity of cyclic *C*-nucleophiles containing two heteroatoms in the ring: barbituric acid (**11**), 1,3-dimethylbarbituric acid (**10**), 2-thiobarbituric acid (**12**) and Meldrum’s acid (**13**). Barbituric acid (**11**) had similar reactivity toward HNO to 1,3-cyclohexanedione (**4**) and dimedone (**6**). In the case of 1,3-dimethylbarbituric acid (**10**), a slight rate enhancement (~fourfold increase over (**11**)) was observed. The reactivity of 2-thiobarbituric acid (**12**) and Meldrum’s acid toward HNO is rather low (*k*_12_ = 8.2 × 10^2^ M^−1^s^−1^, *k*_13_ = 8.7 × 10^2^ M^−1^s^−1^). The corresponding calculated energy barriers for the reaction of barbituric acid (**11**), 1,3-dimethylbarbituric acid (**10**), 2-thiobarbituric acid (**12**) and Meldrum’s acid (**13**) are equal to 64.65, 50.93, 76.28 and 70.15 kJ/mol, respectively.

**Figure 2:**
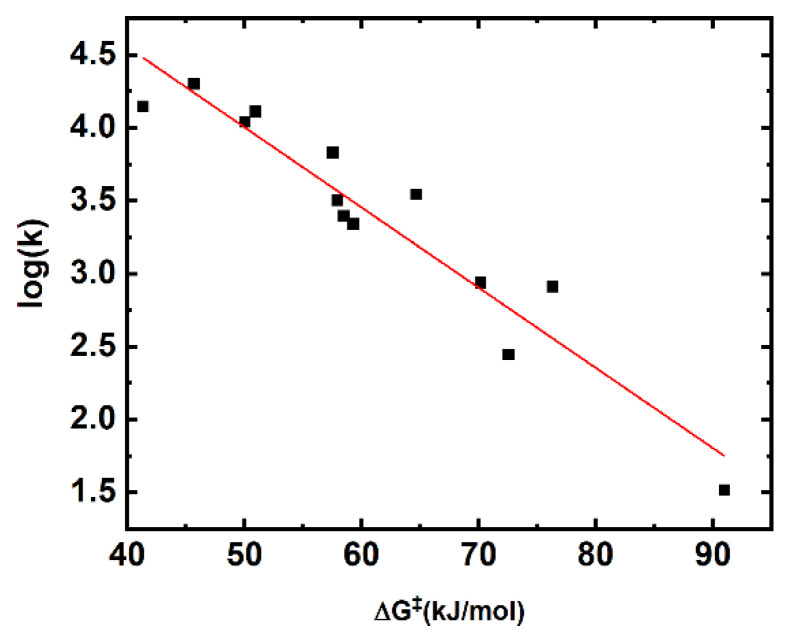
Linear correlation of the computationally determined barrier heights (B2PLYP-D3/6-311+(2df,2p)) versus log(*k*).

Our computationally determined barrier heights correlate linearly with the logarithm of determined rate constants (Figure 2).

Recently, Carrol and Gupta published a study on the reactivity of *C*-nucleophiles toward sulfenic acids [37]. The results of our study show that the reactivity pattern of *C*-nucleophiles towards HNO is similar to that observed for the reaction of *C*-nucleophiles with sulfenic acids.

## 3. Materials and Methods

### 3.1. Equipment

UV-Vis absorption spectra were collected using an Agilent 8453 spectrophotometer equipped with a photodiode array detector and thermostated cell holder.

### 3.2. Chemicals

Synthesis of FlBA boronate probe was described in detail in the Supplementary Materials (Scheme S1 and Figure S1). Angeli’s salt (HNO donor) was synthesized according to the published procedure [38]. The Angeli’s salt stock solution was prepared in 1 mM NaOH. Its concentration was determined by measuring the absorbance at 248 nm (ε = 8.3 × 10^3^ M^−1^cm^−1^) [38]. The solution was kept on ice. 1-(4-Methoxybenzyl)-2,4-piperidinedione, 2-acetyl-1,3-cyclohexanedione were purchased from Angene Chemical. 1,3-Cyclopentanedione, 2-methyl-1,3-cyclopentanedione, 1,3-cyclohexanedione, 2-methyl-1,3-cyclohexanedione, 1,3-cycloheptanedione, and 2,4-piperidinedione were purchased from Fluorochem, United Kingdom. All other chemicals (of the highest purity available) were sourced from Sigma-Aldrich Corp. All solutions were prepared using deionized water (Millipore Milli-Q system).

### 3.3. Kinetic Experiments

The HNO flux was determined from the rate of FlBA oxidation in aerated aqueous solution of Angeli’s salt, monitored at 490 nm (*k* = (8.0 ± 0.1) × 10^−4^ s^−1^). The initial concentration of Angeli’s salt was equal to 20 µM. The calculated initial flux of HNO was therefore close to 0.016 µM/s and was linear during the first 600 s of incubation. Due to the scavenging of HNO by O_2_ and other scavengers, the steady-state concentration of azanone is very low. The HNO dimerization was therefore negligible and was not taken into consideration. Scheme 1 presents a reaction model illustrating the applied competition kinetic method. HNO released from Angeli’s salt reacts either with the HNO scavenger or with the molecular oxygen to form peroxynitrite, which was detected with the use of the FlBA probe (25 µM). Its reaction with ONOO^–^ results in the formation of fluorescein. The formation of fluorescein was monitored spectrophotometrically by following the increase in its characteristic absorbance at 490 nm. The reaction mixtures contained Angeli’s salt (20 µM), the fluorescein-based monoborate probe FlBA (25 µM), phosphate buffer (50 mM, pH 7.4), dtpa (100 µM), and the HNO scavenger (at an appropriate concentration). In addition, each solution contained 5% (vol.) CH_3_CN. The rate constants were determined with the assumption that the concentration of molecular oxygen was equal to 225 µM [39]. Each rate constant was determined in at least three independent experiments.

### 3.4. Computational Details

Quantum mechanical calculations were performed in the Gaussian G09 suite of programs, Revision E01 [40]. Stationary points were found by geometry optimization algorithms with tight convergence criteria except for transition state structure in the reaction of HNO with 1-(4-methoxybenzyl)-2,4-piperidinedione (9) where default criteria had to be used due to lack of computation convergence. To verify the nature of stationary points, as well as to compute Gibbs free energies, respective frequencies were computed. In calculations, the presence of the water environment was described by the Gaussian default continuum solvation model (IEFPCM) [41]. Density functional theory (DFT) functional B2PLYP with Grimme’s D3 dispersion correction [42] (B2PLYP-D3 [43,44]) combined with 6-311+(2df,2p) [45] split valence basis set, was used. The theory level was selected based on the fact that double-hybrid DFT functionals perform well in describing chemical system properties as well as reaction energy barriers, especially when London dispersion corrections are employed [46].

## 4. Conclusions

The results of this study show that *C*-nucleophiles can act as efficient scavengers of HNO. The reactivity of *C*-nucleophiles toward azanone depends strongly on the structure of the scavenger. The reactivity of *C*-nucleophile toward HNO increases with an increase in the ring size. The methylation of α-carbon results in a marked increase in the reactivity, whereas the acylation of this position has the opposite effect. These effects can be attributed to the electronic effects of the substituents. Our results also show, that the alkylation of 1,3-cyclohexanedione at the C-5 position has no effect on the reactivity of this compound. The substitution of one or more ring C-atoms in 1,3-cyclohexanedione with nitrogen results in the increase in *C*-nucleophile reactivity toward HNO. 

Our results also suggest that the reaction of HNO with *C*-nucleophiles can be used in the future for the synthesis of novel HNO donors—*N*-substituted hydroxylamines with carbon-based leaving groups.

## Figures and Tables

**Table 1:** Values of the *k*_S_/*k*_O2_ ratio (average values from at least three independent experiments; pH 7.4, 25 °C) for the studied cyclic *C*-nucleophiles, with the rate constants for their reactions with HNO and theoretically calculated Gibbs free energy barriers at the B2PLYP-D3/6-311+(2df,2p) theory level.

Scavenger	*k*_S_/*k*_Oxygen_	*k*_S_ (M^−1^s^−1^)	ΔG^‡^(kJ/mol)
1,3-Cyclopentanedione (**1**)	0.016 ± 0.001	(2.8 ± 0.6) × 10^2^	72.53
2-Methyl-1,3-cyclopentanedione (**2**)	0.18 ± 0.02	(3.2 ± 0.9) × 10^3^	57.92
2-Acetyl-1,3-cyclopentanedione (**3**)	(1.8 ± 0.2) × 10^−3 [a]^	(3.3 ± 0.8) × 10^1 [a]^	90.93
1,3-Cyclohexanedione (**4**)	0.12 ± 0.01	(2.2 ± 0.4) × 10^3^	59.28
2-Methyl-1,3-cyclohexanedione (**5**)	0.61 ± 0.04	(1.1 ± 0.2) × 10^4^	50.03
Dimedone (**6**)	0.14 ± 0.01	(2.5 ± 0.6) × 10^3^	58.46
1,3-Cycloheptanedione (**7**)	0.38 ± 0.02	(6.8 ± 1.5) × 10^3^	57.52
2,4-Piperidinedione (**8**)	1.13 ± 0.03	(2.0 ± 0.4) × 10^4^	45.65
1-(4-Methoxybenzyl)-2,4-piperidinedione (**9**)	0.80 ± 0.03	(1.4 ± 0.3) × 10^4^	41.34
1,3-Dimethylbarbituric acid (**10**)	0.72 ± 0.03	(1.3 ± 0.3) × 10^4^	50.93
Barbituric acid (**11**)	0.20 ± 0.01	(3.5 ± 0.8) × 10^3^	64.65
2-Thiobarbituric acid (**12**)	0.046 ± 0.003	(8.2 ± 1.9) × 10^2^	76.28
Meldrum’s acid (**13**)	0.048 ± 0.002	(8.7 ± 1.8) × 10^2^	70.15

^[a]^ The *k*_S_/*k*_O2_ ratio was evaluated based on the effect of 10 mM 2-acetyl-1,3-cyclohexanedione (**3**) on the FlBA oxidation rate.

## Data Availability

The data presented in this study are openly available in open-access repository Zenodo at doi:10.5281/zenodo.5736579.

## Supplementary Materials

The Supplementary Materials are available online at www.mdpi.com/article/10.3390/ijms222312982/s1.
