# Supplementary material for: Kinetic Study on the Reactivity of Azanone (HNO) toward Cyclic C-Nucleophiles
Source: Int J Mol Sci. 2021 Nov 30;22(23):12982. doi: 10.3390/ijms222312982 (PMC8657990; doi:10.3390/ijms222312982)
Supplement: Supplementary file 1 [file ijms-22-12982-s001.zip › ijms-1474019-supplementary.pdf]

Kinetic Study on the Reactivity of Azanone (HNO) toward
Cyclic *C*-Nucleophiles

Angelika Artelska, Monika Rola, Michał Rostkowski, Marlena Pięta, Jakub Pięta, Radosław Michalski and Adam Bartłomiej Sikora *

Institute of Applied Radiation Chemistry, Lodz University of Technology, Zeromskiego 116,
90-924 Lodz, Poland; angelika.artelska@dokt.p.lodz.pl (A.A.);
monika.rola@dokt.p.lodz.pl (M.R.);
michal.rostkowski@p.lodz.pl (M.R.); marlena.pieta@p.lodz.pl (M.P.);
jakub.pieta@p.lodz.pl (J.P.);
radoslaw.michalski@p.lodz.pl (R.M.)
* Correspondence: adam.sikora@p.lodz.pl

1. Synthesis of Fluorescein Boronic Acid (FlBA)

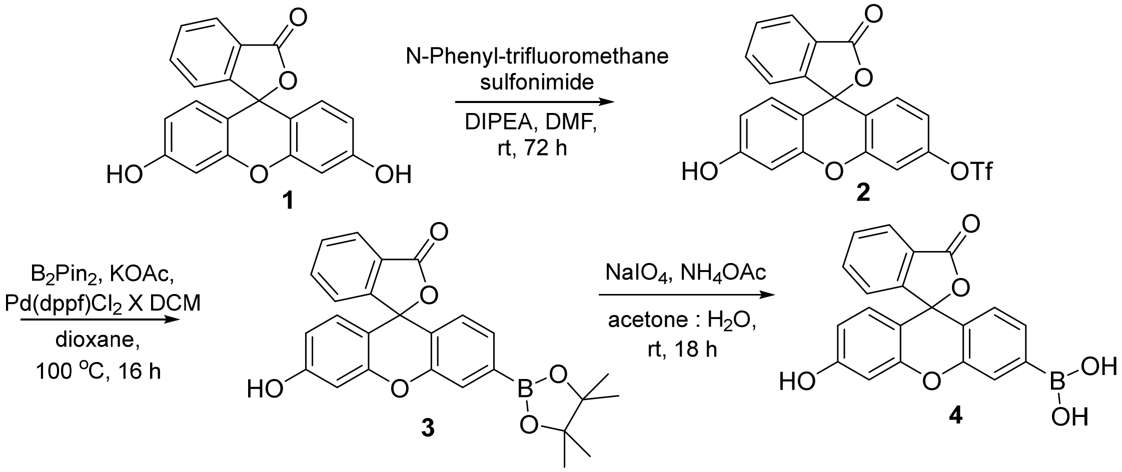

Scheme S1. Synthesis of Fluorescein Boronic Acid (FlBA).

Two first steps of synthesis were performed based on procedures described in the literature.[1,2] Solution of fluorescein **1** (1.0 g, 3.01 mmol), *N*-phenyl-bis(trifluoromethanesulfonimide) (1.075 g, 3.01 mmol) and DIPEA (2.0 mL, 14.3 mmol) in DMF (10 mL) was protected from light and stirred at rt under argon atmosphere for 72 hours. Then 10 mL of 1M HCl was added and the resulting mixture was extracted with AcOEt (3 × 25 mL), dried over $MgSO_4$ and condensed. After purification by column chromatography (hexane/AcOEt from 10:0 to 10:1) 1.40 g of fluorescein triflate **2** was obtained (65 % yield). ESI-MS m/z $[M+H]^+$ = 465.03.

Then triflate **2** (900 mg, 1.94 mmol), bis(pinacolato)diboron (700 mg, 2.76 mmol), $Pd(dppf)Cl_2$*CH_2Cl_2 (160 mg, 0.196 mmol) and KOAc (634 mg, 6.12 mmol) were placed in screw cap glass vial and suspended in dioxane (4 mL). It was purged with argon, closed and stir at 100 °C for 16 hours. After cooling to rt the mixture was diluted in AcOEt and washed with water and brine, dried over Na_2SO_4 and condensed. After purification by column chromatography (hexane/AcOEt from 19:1 to 7:3) 600 mg of fluorescein pinacol boronester **3** was obtained (70 % yield). ESI-MS m/z $[M+H]^+$ = 443.16.

Fluorescein pinacol boronester **3** (300 mg, 0.678 mmol), sodium periodate (1,16 g, 5.43 mmol) and ammonium acetate (418 mg, 5.43 mmol) were suspended in acetone (12 mL) and H_2O (12 mL).

The mixture was stirred at rt overnight. The acetone was evaporated and the residue extracted with AcOEt (4*15 mL). Organic layer was dried over $MgSO_4$, condensed and separated by column chromatography ($CHCl_3$/MeOH from 19:1 to 9:1) to give 210 mg (86% yield) of desired product **4** as orange solid. R_f=0.23 (Hexane/AcOEt 7:3); ^{1}H NMR, (700 MHz, CD_3OD): δ 8.01 (dd, $^3J_{H,H}$ = 7.6 Hz, 1H, Ar-H), 7.76 (dt, $^3J_{H,H}$ = 7.6, $^4J_{H,H}$ = 1.1 Hz, 1H, Ar-H), 7.71 (dt, $^3J_{H,H}$ = 7.6, $^4J_{H,H}$ = 1.0 Hz, 1H, Ar-H), 7.53 (bs, 1H), 7.29 (bd, $^3J_{H,H}$ = 6.8 Hz, 1H, Ar-H), 7.20 (d, $^3J_{H,H}$ = 7.6 Hz, 1H, Ar-H), 6.76 (bd, $^3J_{H,H}$ = 6.2 Hz, 1H, Ar-H), 6.72 (d, $^4J_{H,H}$ = 2.4 Hz, 1H), 6.61 (d, $^3J_{H,H}$ = 8.7 Hz, 1H), 6.55 (dd, $^3J_{H,H}$ = 8.7, $^4J_{H,H}$ = 2.4 Hz, 1H). ESI-MS m/z $[M+H]^+$ = 361.09.

1. Dickinson, B.C.; Huynh, C.; Chang, C.J. A palette of fluorescent probes with varying emission colors for imaging hydrogen peroxide signaling in living cells. *Journal of the American Chemical Society* **2010**, *132*, 5906-5915, doi:10.1021/ja1014103.
2. Rios, N.; Piacenza, L.; Trujillo, M.; Martínez, A.; Demicheli, V.; Prolo, C.; Álvarez, M.N.; López, G.V.; Radi, R. Sensitive detection and estimation of cell-derived peroxynitrite fluxes using fluorescein-boronate. *Free radical biology & medicine* **2016**, *101*, 284-295, doi:10.1016/j.freeradbiomed.2016.08.033.

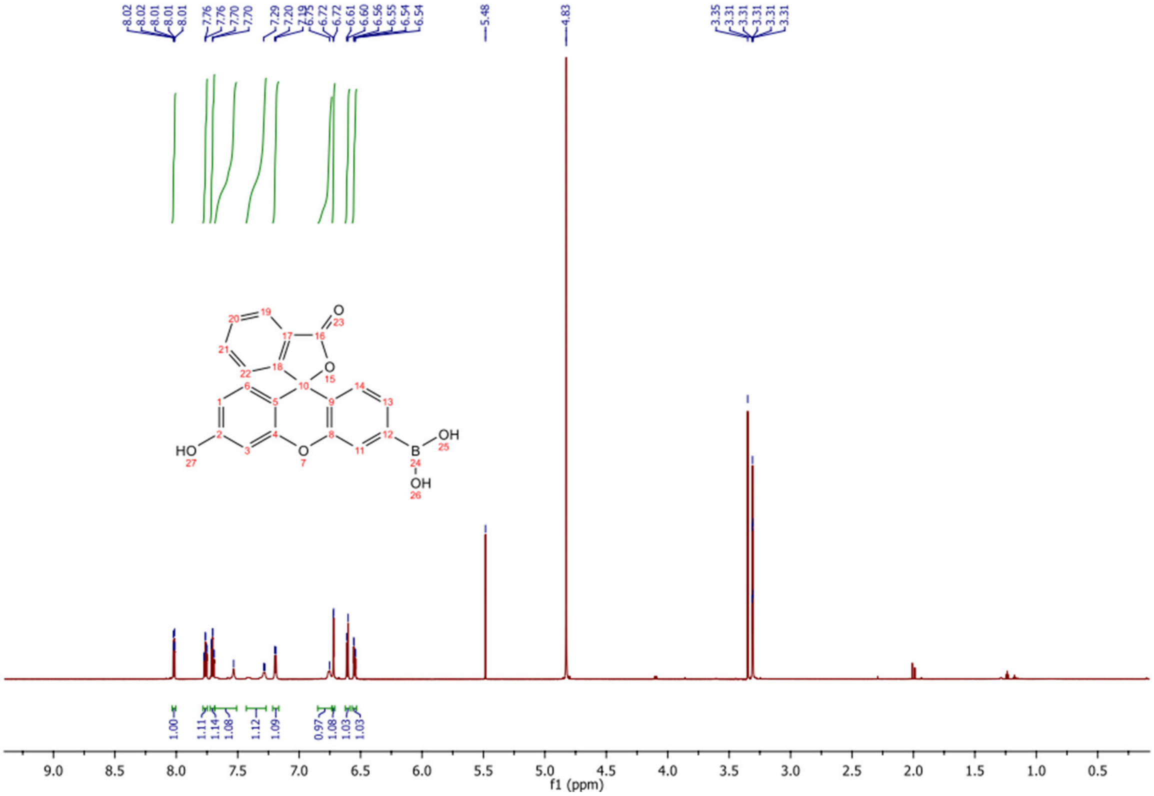

Figure S1. 1H MNR spectrum of FlBA probe solution in CD_3OD.

2. Reactivity of Fluorescein Boronic Acid (FlBA) Toward Peroxynitrite

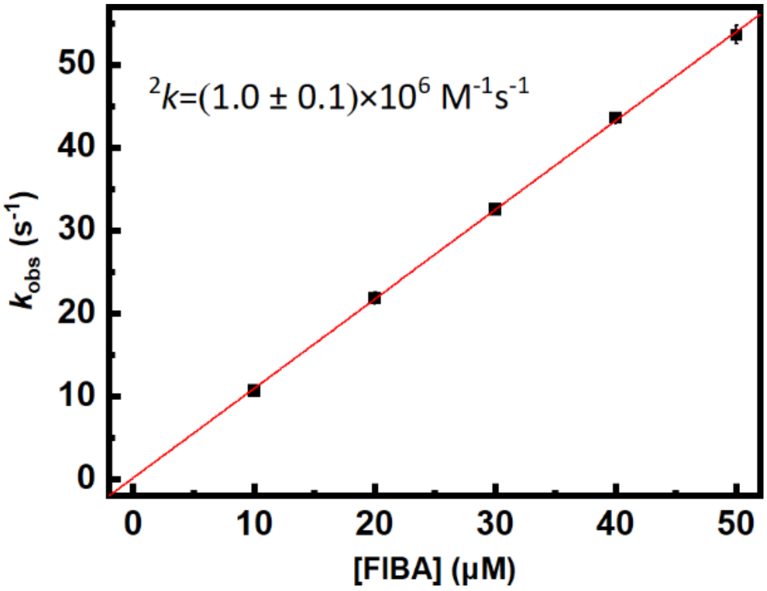

Figure S2. The dependence of the pseudo-first order rate constant of the reaction of peroxynitrite with the boronate probe FlBA. The reaction mixture contained: 10-50 μM FlBA, 2 μM $ONOO^-$, 50 mM phosphate buffer (pH 7.4), 10% vol. CH_3CN. The formation of fluorescent fluorescein was monitored using an Applied Photophysics SX20 stopped-flow spectrophotometer equipped with a fluorescence detector and a thermostatically controlled cell (25 °C) with a 10-mm optical pathway. The reaction mixtures were excited at 492 nm (PMT voltage = 600 V, emission/excitation slit = 2.5 nm).

3. Reactivity of Fluorescein Boronic Acid (FlBA) Toward H2O2

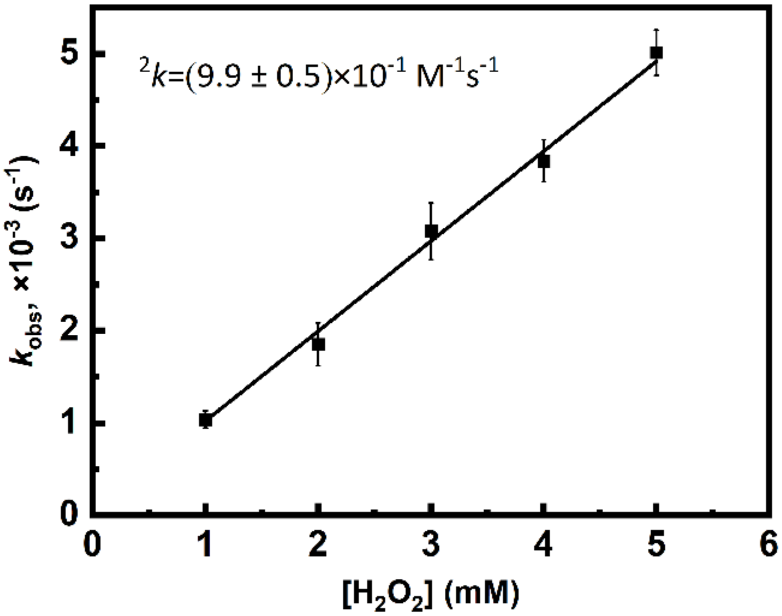

Figure S3. The dependence of the pseudo-first order rate constant of the reaction of hydrogen peroxide with the boronate probe FlBA. The reaction mixture contained: 20 μM FlBA, 1-5 mM H_2O_2, 50 mM phosphate buffer (pH 7.4), 5% vol. CH_3CN. The formation of fluorescein was monitored using an spectrophotometer at a wavelength of 490 nm.
